# The Impact of Information Sources on COVID-19-Related Knowledge, Attitudes, and Practices (KAP) among University Students: A Nationwide Cross-Sectional Study

**DOI:** 10.3390/ijerph182312462

**Published:** 2021-11-26

**Authors:** Israa Baker, Nizar Marzouqa, Bashar Nafe’ Yaghi, Samer Osama Adawi, Shahd Yousef, Tayseer Nedal Sabooh, Nataly Mazen Salhab, Hiba Mahmoud Khrishi, Yahya Qabaja, Abanoub Riad, Elham Kateeb, Sameh Attia

**Affiliations:** 1 Al-Quds Medical Research Association, Faculty of Medicine, Al-Quds University, Jerusalem 510 00, Palestine; israa.baker@students.alquds.edu (I.B.); nizar.marzouqa@students.alquds.edu (N.M.); bashar.yaghi@students.alquds.edu (B.N.Y.); samer.adawi@students.alquds.edu (S.O.A.); shahd.yousef@students.alquds.edu (S.Y.); taysir.saboh@students.alquds.edu (T.N.S.); nataly.salhab@students.alquds.edu (N.M.S.); hibakh.393@gmail.com (H.M.K.); yahaya.qubaja@students.alquds.edu (Y.Q.); 2 Department of Public Health, Masaryk University, 625 00 Brno, Czech Republic; abanoub.riad@med.muni.cz; 3 Oral Health Research and Promotion Unit, Al-Quds University, Jerusalem 510 00, Palestine; 4 Department of Oral and Maxillofacial Surgery, Justus-Liebig-University, 353 92 Giessen, Germany

**Keywords:** knowledge, information sources, information checking, COVID-19, Palestine

## Abstract

COVID-19 is an emerging respiratory disease caused by a novel coronavirus accompanied by a tsunami of misinformation and fake news. This can weaken the public health responses by affecting the COVID-19-related knowledge, attitudes, and practices (KAP) of the public. Therefore, this cross-sectional study was designed during the early stage of the pandemic to evaluate the KAP of Palestinian university students and their commonly used information sources. We found that the most trusted information source among students was the World Health Organization (WHO), followed by the Palestinian Ministry of Health (MoH) briefings and healthcare workers, whereas social media was the most frequently used source of information. The participants exhibited a high level of COVID-19-related knowledge, having an average score of 8.65 (range: 0–10). In total, 76% avoided going to crowded places, and only 33% wore a mask while being outdoors. The vast majority (93%) checked the accuracy of COVID-19-related information before publishing it, 56% used the WHO and MoH briefings for fact-checking, and only 8% relied on healthcare workers. This was particularly the case for those who lived in refugee camps. This study provides an insight into the information sources used by Palestinian university students, the sources they trust, and the information formats they prefer. These results may help public health authorities to locate the information sources through which university students should be targeted. Efforts should be made to recommend healthcare workers as credible information sources. In this way, they will be able to prevent the spread of misleading information and provide high-quality information, especially within unconventional settings such as refugee camps.

## 1. Introduction

On 30 December 2019, the Chinese health authorities notified the World Health Organization (WHO) of the first cohort of cases with “pneumonia of unknown etiology” in Wuhan City, Hubei Province. Five days later, the WHO declared that there was an infectious disease in central China [1]. On 7 January 2020, the Chinese authority isolated the causative agent, which was a member of the *Coronaviridae* family [2]. The WHO announced the official names of the virus and the resulting infectious disease on 11 February 2020: the severe acute respiratory syndrome coronavirus-2 (SARS-CoV-2) and the coronavirus disease 2019 (COVID-19), respectively. On 11 March 2020, COVID-19 was officially declared a pandemic, and all of the world’s governments were called upon to act accordingly [3]. 

Coronaviruses are enveloped positive-sense, single-stranded RNA viruses. They most commonly cause mild respiratory tract infections. However, some forms can be lethal, such as SARS-CoV-2, which has an incubation period of 2 to 14 days, with a median of approximately 5.1 days [4]. Many cases are asymptomatic, but if symptoms emerge, the most common are lower respiratory tract symptoms, including fever, shortness of breath, and non-productive cough. Other systems may also be affected, e.g., the gastrointestinal system, with symptoms such as nausea, vomiting, and diarrhea; and the nervous system, with symptoms such as anosmia and dysgeusia [5,6,7,8,9,10,11,12,13]. Moreover, dermatologic and oral symptoms have been consistently reported among COVID-19 patients with an overwhelming frequency and a lack of a clear pathophysiologic pathway [11,14,15,16,17,18]. SARS-CoV-2 is predominantly transmitted via respiratory droplets from coughing or sneezing, especially in closed spaces, and indirect contact by touching surfaces and objects in the immediately surrounding environment of an infected person [4,19]. 

Although many clinical trials were underway globally for the treatment of COVID-19 for a number of months, no antiviral drugs were clinically approved by the Food and Drug Administration (FDA) for the treatment of COVID-19 until recently [20]. In October 2021, the early results of a phase 2/3 clinical trial revealed that Molnupiravir had a protective effect against hospitalization and mortality due to COVID-19 [21]. In addition, on 3 December 2020, the WHO authorized the Pfizer-BioNTech vaccine for emergency use for COVID-19 [22]. Additionally, the Oxford-AstraZeneca COVID-19 vaccine began to roll out in the first quarter of 2021 [23]. As of September 2021, more than 5.98 billion shots of COVID-19 vaccines had been administered in nearly all countries, with substantial levels of safety and acceptable effectiveness [24,25,26,27,28,29,30]. Prior to the vaccines’ authorization, recovery relied entirely on a well-functioning immune system [4]. Therefore, the focus has moved towards prevention, where many countries have applied wide-ranging quarantines, travel restrictions, sick isolation, contact-tracing, and physical distancing measures to limit the spread of the disease and reduce the number of cases [31].

Several epidemiological studies have shown that quarantine policies have reduced COVID-19’s transmission and helped “flatten the curve” of active cases, leading to less pressure on the health systems, and higher availability of ventilators, testing kits and intensive care unit beds, in addition to reduced mortality and slower economic deterioration [32,33,34]. Unfortunately, this may also have several financial, psychological, and social disadvantages, affecting the already unconventional populations [33,35,36]. 

These anti-pandemic measures had adversely affected universities and mandated teaching methods to move online; however, many study programs, including healthcare programs, were not ready for this abrupt shift [35,36,37,38]. University students are widely depicted as opinion leaders within their local communities when it comes to health-related issues due to their supposedly high levels of health literacy; therefore, their health-related beliefs, knowledge and attitudes are of practical value for primary prevention interventions [39,40,41,42]. Students and academics were severely affected by these measures in various aspects including their attitudes, stress-coping, and information-seeking strategies [43,44,45,46,47].

The COVID-19 pandemic started in Palestine on 5 March 2020 in Bethlehem city [48]. Hence, the Palestinian government declared a nationwide state of emergency on the same day [48]. In the following months, Palestinian ministers held multiple public press briefings on the updates of the pandemic situation in Palestine, and accordingly implemented movement restrictions in different governorates [49]. As of 16 November 2021, the Palestinian Ministry of Health (MoH) reported 457,479 COVID-19 cases in Palestine, 2946 of which were active, and 4764 deaths [50]. 

With many unanswered questions, it was easy for misinformation to circulate. A trend of “fake news” was flooding social media platforms with fabricated scientific information on the virus’s transmission, origins and prevention [48,51]. Not only do media outlets play a major role in explaining the details of a crisis, but they can also sway the public’s political and social views and influence their beliefs [52]. The WHO had identified the pandemic’s risk to Palestine as “very high”, mainly due to the lack of medical support [50]. Therefore, the public’s adherence to specialists’ recommendations was of particular importance. 

The needed cooperation between individuals relies on their belief in the collective efficacy of each person changing their practices [53]. For example, one study linked understanding the COVID-19 situation to better adherence to health-protective practices, with variable effects of the information sources used [54]. This was also evident during the swine flu (H1N1) pandemic in 2009, when uncertainty and mistrust in sources of information, both formal and informal, was linked to lower compliance rates with public health instructions [55,56].

Several studies have been conducted during the ongoing COVID-19 pandemic to tackle misinformation [57]. Some have shown that the need for information in times of crises combined with the public nature of social media are important reasons for the spread of inaccurate information [58]. Most fabrication revolved around COVID-19’s statistics, origins, transmission, prevention and treatment [58,59]. False narratives can affect people’s health and safety, as is the case of mistrusting mask regulations or the false rumors surrounding hydroxychloroquine’s therapeutic efficacy [59,60]. Furthermore, this atmosphere of mistrust can lead to negative psycho-social outcomes with economic and ethical healthcare considerations [58]. Recent studies suggest that dealing with misinformation requires a multidisciplinary approach, of which key steps are identifying fake news, tackling misinformation, and widely sharing accurate information [60,61].

As such, effective COVID-19 control necessitates studying information-seeking patterns and their effect on society’s perceptions and practices, focusing on the susceptible groups for misinformation, e.g., male college students. In an effort to do so, this study was designed to assess the knowledge, attitudes, and practices (KAP) of university students in Palestine towards COVID-19 and their common and trusted information sources.

## 2. Materials and Methods

### 2.1. Design 

This cross-sectional study was conducted from 9 to 25 May 2020, and targeted bachelor level (undergraduate) university students aged from 18 to 30 years. Seven Palestinian universities were randomly selected to represent the major governorates in the West Bank and Gaza, with a priority for the universities with healthcare programs.

The selected universities were Al-Quds University (Jerusalem), Al-Najah University (Nablus), Birzeit University (Ramallah and Al-Bireh), Al-Azhar University (Gaza), Bethlehem University (Bethlehem), Arab American University (Jenin), and Palestine Polytechnic University (Hebron).

### 2.2. Participants

According to the Palestinian Ministry of Higher Education (MHE), the total number of students enrolled in the seven target universities during the academic year 2018/2019 was 74,367. The optimal sample size required for this study was calculated using the Creative Research Systems (CRS) online calculator with a confidence level of 99% and a margin of error of 5% [62]. The sample size needed was 660 subjects. To ensure good representation of all universities genders and disciplines, data collection was based on proportionate stratified random sampling according to each university actual percentage [62]. 

### 2.3. Instrument

A self-administered questionnaire (SAQ) was developed and circulated online to collect data from the target participants. The SAQ consisted of multiple-choice items stratified over three main categories: (a) demographic information, (b) information sources, and (c) COVID-19-related knowledge, attitudes, and practices (KAP). The average time needed to provide the consent form and complete the SAQ questions was ten minutes. The identity of the respondents remained anonymous.

The questions were adapted from previous questionnaires that were initially written in English then translated into Arabic [63]. The content validity was carried out by four experts in the fields of medical and public health, who reviewed and gave their feedback for the drafted questions.

The first category, “demographic information”, inquired about the students’ age, gender, university, study program, academic year, place of residence, and monthly household income in Israeli new shekel (ILS).

The second category, “information source”, included a suggested list of the frequently used sources, the most trusted source, the most used social media platforms for information acquisition, the preferred type of information format, the interest in COVID-19-related information updates, and the fact-checking mechanisms for the acquired information. In addition, we added a few questions about the students’ health status in regards to COVID-19, and if they knew someone who was infected with COVID-19. 

The third category, “COVID-19-related KAP”, included questions about knowledge (*n* = 10), attitudes (*n* = 3), and practices (*n* = 3), which were mostly Yes/No questions, except for one multiple-choice question. A higher KAP score indicated sound knowledge, cautious attitudes, and good practices. The information was rechecked using U.S. Food and Drug Association (FDA), Mayo Clinic, and the World Health Organization (WHO) [64,65,66]. 

### 2.4. Data Collection

The SAQ was developed and circulated online using Google Forms (Google LLC, Mountain View, CA, USA) due to the physical distancing recommendations set by the Palestinian Ministry of Health (MoH). The SAQ was distributed through Facebook groups of the students enrolled in the target universities, and instant messaging applications, i.e., Messenger (Facebook Inc., Menlo Park, CA, USA) and WhatsApp (Facebook Inc., Menlo Park, CA, USA).

To ensure good representation of the target population, the sample size portions were reviewed at intervals of 150 respondents to remove missing or excluded data directly, follow up percentages, and adjust data collection. Accordingly, of the initially collected 1110 responses, 159 were excluded due to various reasons: 39 from other universities, 22 outside the age group, 93 postgraduate students, and 5 missing data.

### 2.5. Outcome Measures

To measure the COVID-19-related knowledge level, a simple addition of scores of answers to the ten knowledge questions was used, with a Cronbach’s alpha of 0.62, which indicated a poor, yet acceptable, scale internal consistency [67,68]. The answers were assigned a number of points based on the previous literature [69]. Three COVID-19 attitudes questions and three COVID-19-related practices questions were studied individually. 

The information sources used were collected in two ways; the first was from a question with a “check all that applies” feature, and the second asked about the single most used information source. They were used individually for descriptive purposes and combined in subgroups for analysis (Table 1).

### 2.6. Ethical Considerations

The study was designed and carried out according to the Declaration of Helsinki for research involving human subjects, and was reported according to the Strengthening the Reporting of Observational Studies in Epidemiology (STROBE) guidelines for cross-sectional studies [70,71]. The study protocol was reviewed and approved by Al-Quds University Research Ethics Committee. Prior to their participation in the study, all the participating students had to provide their digital informed consent, which included information about the study purpose, data anonymity, and voluntary nature of the participation.

### 2.7. Statistical Analysis

The Statistical Package for the Social Sciences (SPSS) version 27 (SPSS Inc., Chicago, IL, USA, 2020) was used to perform all the statistical tests [72]. Primarily, descriptive statistics analysis was performed to summarize the acquired sample characteristics and outcomes, where frequencies (*n*) and percentages (%) were used to represent categorical variables, and means (*µ*) and standard deviations (*SD*) were used with numerical variables.

Subsequently, inferential statistics analysis was performed using Student’s *t*-test, analysis of variance (ANOVA), and Pearson’s chi square test (*χ*^2^) to test the hypothesized associations between the study variables. Whenever the ANOVA test was significant, Turkey’s post hoc test was used to check for intra-variable significant differences. The statistical differences were considered significant when the *p*-value was ≤0.05.

## 3. Results

### 3.1. Demographic Characteristics

A total of nine hundred and fifty-one students completed the online survey properly thus constituting a crude response rate of 6.25%. The majority of the participants were females (69.8%), aged 23 years or below (95.2%), single (96.6%), and enrolled in non-healthcare programs (64.6%). Although 57.7% were from urban areas, the remainder were from rural areas (35.4%) and camps (6.8%). More than half of the participating students (54.6%) reported that their monthly household income was less than ILS 4000 (Table 2).

**Figure 1:**
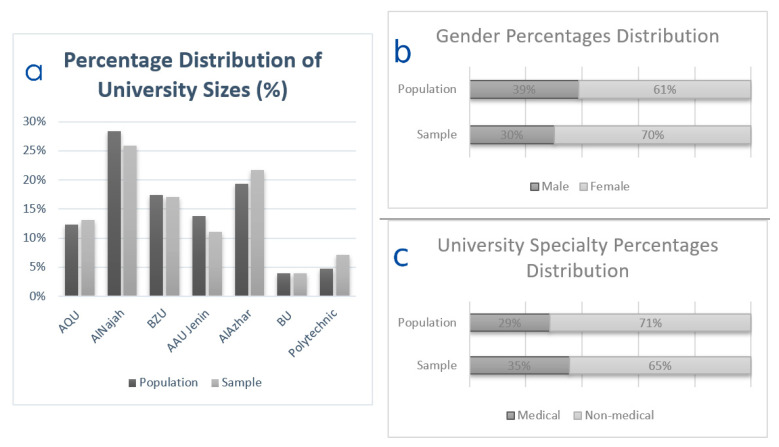
Response rate and representativeness of the participating students: (**a**) response rate; and (**b**) representativeness by gender and (**c**) by study program.

On evaluating the representativeness of our acquired sample, the female-to-male ratio in our sample (69.8%:30.2%) was a similar to the target population of university students in Palestine (61%:39%). Moreover, our sample reflected the current distribution of the students by study programs, as 35.4% of our participants were healthcare students and 29% of the total students in the surveyed universities were also enrolled in healthcare programs (Figure 1).

### 3.2. Information Sources

**Figure 2:**
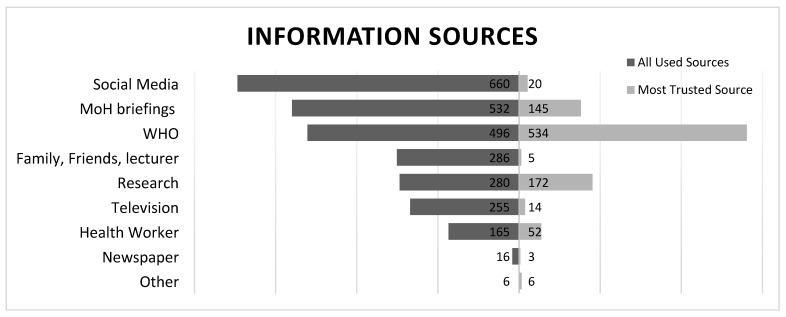
The most frequently used information sources and the most trusted sources. Note that the sum of the information sources used does not equal 951 (the total population), because it was calculated by a “check-all-that-applies” question, so a respondent who chose multiple answers would be counted multiple times. The most trusted source was a one-answer-only question.

In general, the WHO was the most trusted information source (56.2%) followed by scholarly articles (18.1%) and MoH briefings (15.2%), whereas the most frequently used information source was social media platforms (69.4%) followed by MoH briefings (55.9%) and the WHO (52.2%) Figure 2.

**Figure 3:**
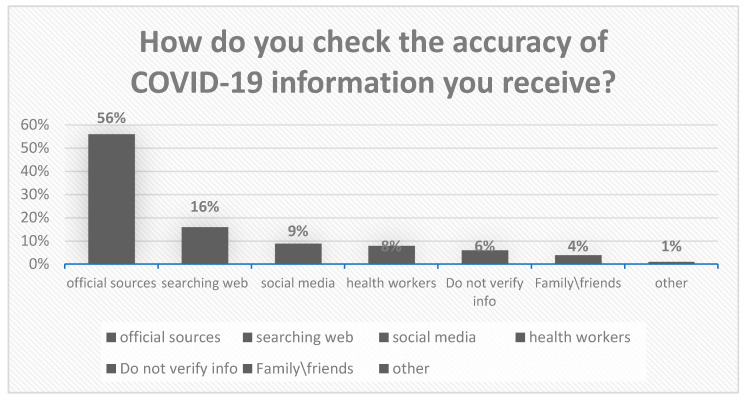
Distribution of the sources used for checking the accuracy of COVID-19-related information.

Most respondents (93.5%) fact-checked COVID-19 information before publishing it, the majority (56%) used official sources to do so. In regard to social media and health workers as a source of information, 9.2% used social media to check the accuracy of COVID-19 information, and only 7.6% of the participants checked with a healthcare worker. As for information type, most students used sources that present information in text (59.1%) followed by video (28.3%) (Figure 3).

There was no significant difference in information sources across the demographic variables except for the study field (*χ*^2^ = 4 × 10^7^; *p* = 0.06). The source most frequently used by the healthcare students (18.1%) was a combination of official, scientific, and community sources, whereas only the social media platforms sources were the most common among non-healthcare students (18.4%). 

Similarly, there were significant associations between the students’ most trusted sources and their study program (*χ*^2^ = 36.2; *p* < 0.001), social status (*χ*^2^ = 50; *p* = 0.03), and the place of residence (*χ*^2^ = 36.4; *p* = 0.003). In general, WHO as the most trusted source was more preferred by the healthcare students (66%) than non-healthcare students (51%). Interestingly, the MoH briefings were most trusted by the students who lived in the rural areas (19%), whereas healthcare workers (14%) were the most trusted by students from camps.

### 3.3. COVID-19-Related Knowledge

The results indicated that the overall COVID-19-related knowledge is moderate, with correct answers amounting to 82.5% of the total. The majority of the participants were aware of the most common symptoms of COVID-19 and 67.5% of them were cognizant of unusual symptoms such as nasal congestion and diarrhea. The vast majority (94.2%) knew that COVID-19 spreads among people through droplets released from the mouth or nose when an infected person coughs or exhales.

In contrast, 61% did not know whether the virus can be transmitted in areas with hot and humid climates, 62% did not know whether the virus can be transmitted through mosquito bites, and only 8% of students knew that infected people can infect others even if they were asymptomatic. There was no significant difference in knowledge level across the demographic variables (Table 3).

### 3.4. COVID-19-Related Attitudes

Most of the participants were concerned about the impact of this epidemic on the Palestinian community (82%). There was a significant relationship between the concern about outbreak impact and the place of residence (*χ*^2^ = 10; *p* = 0.04), as the students living in refugee camps were most anxious (6.8%). There was no significant difference in attitudes across the other demographic variables (Table 4).

### 3.5. COVID-19-Related Practices

Most students did not go to crowded places during the periods of lockdown or restricted movement and human interaction that the MoH recommended; however, few students wore masks when leaving their houses. Wearing a mask was significantly associated with gender (*χ*^2^ = 8; *p* = 0.03) and academic year (*χ*^2^ = 20; *p* = 0.03). The percentage of those who, in that period, wore a mask when they left their homes was higher among females (34.9%) than males (27.5%) (Table 5).

### 3.6. Impact of Information Sources on COVID-19-Related KAP

Information sources were not significantly associated with the COVID-19-related knowledge subscale (*p* = 0.08). There were no significant differences in socializing with people and going to crowded places based on information source used, but there was a significant association between wearing a mask when leaving the house and the information source (*p* = 0.05). Amongst the information sources used by >2% of the target population, those who used official and scientific sources together made up an equal percentage as students who wore a mask outside the house (50%), whereas the highest percentage of these not wearing the mask were students who used the community as an exclusive source Table 6.

### 3.7. Information Seeking Strategies and COVID-19 Knowledge

The information sources that the students found credible significantly affected their knowledge (*χ*^2^ = 14.7; *p* < 0.001). Significant differences (post hoc; *p* < 0.05) in the mean knowledge score were found between MoH briefings (8.1), healthcare workers (7.7), social media platforms (7.8), scholarly articles (8.9), and the WHO (8.9). 

There was a significant difference in the mean knowledge score across the mechanisms the students used to check facts related to COVID-19 (*p* = 0.001). The students who asked family and friends for verifying information (7.7) were significantly (post hoc; *p* < 0.05) less knowledgeable compared to those who checked using social media (8.8), official sources (8.7), and search engines (8.7).

### 3.8. Information Seeking Strategies and Attitudes towards COVID-19

The concern over the pandemic impact was associated with social media as the most frequently used information source (*χ*^2^ = 28; *p* = 0.005), checking information before publishing (*χ*^2^ = 6.2; *p* = 0.045), method of checking (*χ*^2^ = 26; *p* = 0.01), and knowing infected people (*χ*^2^ = 37.4; *p* < 0.001). The most concerned students were those that used Snapchat the most (100%), followed by Instagram (93%) and WhatsApp (89%).

Those who checked the accuracy of COVID-19 information before publishing (83%) were more concerned than those who did not (69%). Those who checked by official sources were the most concerned (86%), whereas the least were those who checked by “other” (64%). Those who had distant relatives with COVID-19 were more concerned (84%) than those who had a close infected relative (80%), an infected friend (71%), or knew no-one with COVID-19 (82%).

Checking information was significantly associated with the thought that public health services were well prepared for COVID-19, whereas those who did not check were slightly more optimistic (55%) than the ones who did (50%). The students’ belief in COVID-19 pandemic containment was significantly associated with their most trusted source (*p* = 0.003), their method of checking (*p* = 0.009), and their preferred media platform (*p* = 0.049) Table 7.

### 3.9. Information Seeking Strategies and COVID-19-Related Practices

The students’ most trusted source of COVID-19 information significantly affected their socialization patterns, e.g., going to crowded places (*χ*^2^ = 42; *p* = 0.003) and wearing a mask while being outdoors (*χ*^2^ = 36; *p* = 0.003).

**Figure 4:**
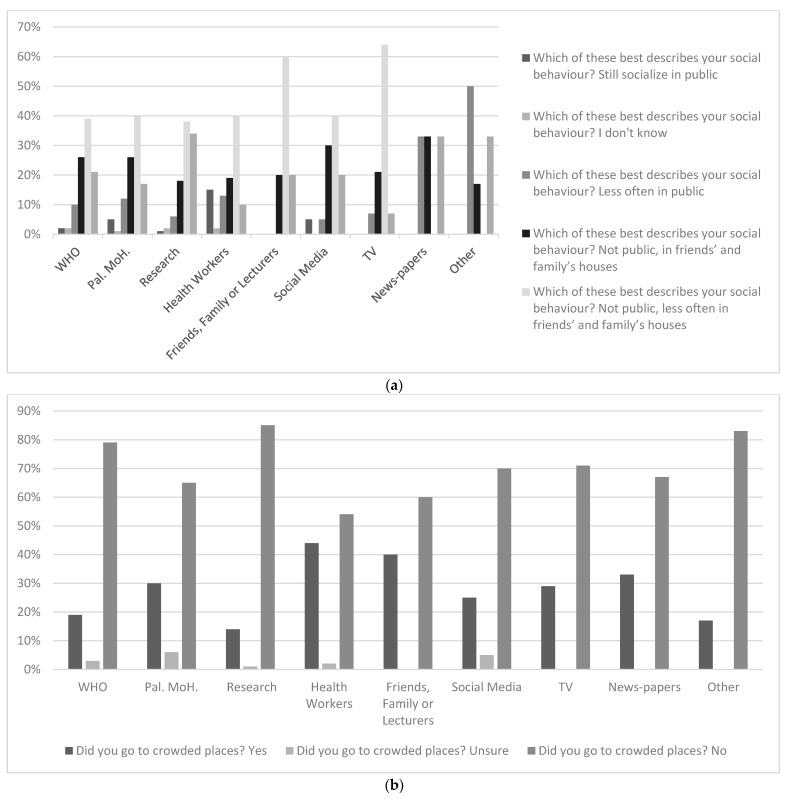

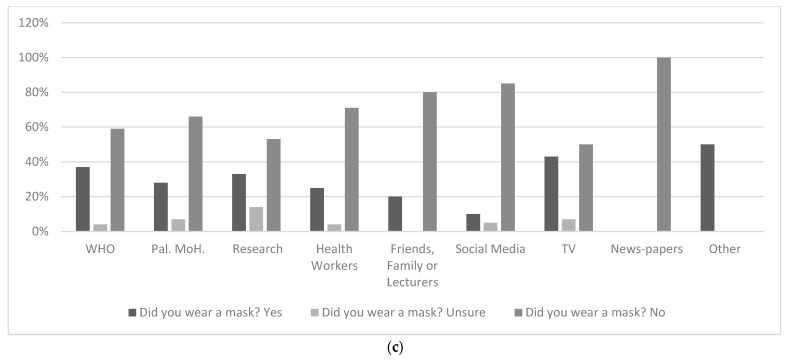
Information-seeking strategies and COVID-19-related practices: (**a**) general behaviors, (**b**) socialization patterns, and (**c**) wearing masks.

Among those who chose one preferred COVID-19 information format, students who chose voice were the most inclined to wear masks while being outdoors (47%), followed by those who preferred the video format (39%) Figure 4.

## 4. Discussion

The COVID-19 pandemic is a serious global health threat, caused by a novel coronavirus and accompanied by a tsunami of misinformation and fake news. This is the first study to explore the accuracy of COVID-19-related KAP and the information-seeking strategies among the Palestinian population. With a sample of 951 undergraduate university students from several study programs, and in multiple universities distributed in the West Bank and the Gaza strip, this study evaluated the most frequently used and the most trusted information sources, in addition to the methods that the youth used in order to verify the information related to COVID-19.

This comprehensive study found that the most frequently used information source was social media. Interestingly, these results are consistent with previous studies conducted among students of Jordanian universities, and the general Egyptian population [73,74,75]. This high social media use was expected, as the non-medical numbers were approximate twice more than medical students. According to the data comparison among the study fields, medical students frequently utilize official, scientific, and community sources, whereas non-medical students use social media. Facebook was the most accessed platform for social media users. Similarly, Abdelhafiz et al. 2020 reported that Facebook was the continual social media platform among Egyptian public residents, noting that 75% of participants aged 18–40 years made use of the platform [75]. In addition, the Mixed Migration Center has published a similar finding among Afghan returnees [76]. As Facebook is an easy-access online application and a worldwide platform, it is worth mentioning that it is beneficial for spreading information regarding COVID-19 [77]. However, fact-checking this information is essential to avoid spreading fake news regarding COVID-19. Surprisingly, 90% of our participants re-checked their information mainly from official sources, such as the World Health Organization and the Palestinian Ministry of Health. As predicted, WHO represents the primary credible source of information. Interestingly, the Ministry of Health was the most trusted by people in villages.

The results of this study revealed that the students were knowledgeable about the most common symptoms of COVID-19 (97%) such as fever, tiredness, and dry cough, and even the less common symptoms (91%) such as pain, nasal congestion, and diarrhea. The WHO reported that the most common symptoms of COVID-19 were fever, dry cough, and tiredness [78]. The less common symptoms include aches and pains, nasal congestion, headache, conjunctivitis, sore throat, diarrhea, loss of taste or smell, a rash on the skin, and discoloration of fingers or toes. It is worth mentioning that the lower knowledge score was related to the transmission questions; only 8% knew that persons with COVID-19 are infectious even if they were asymptomatic [79].

The concerns over the COVID-19 outbreak in Palestine were significantly higher among the students who lived in refugee camps compared to those who live in urban and rural areas. The main factor is the way in which camps are structured, as they have limited spaces with poor ventilation and crowded buildings, and are densely populated; therefore, the chance of the disease spreading is very high [80,81].

The participating students were mostly aware of the WHO recommendations, in addition to the Palestinian government undertaken measures and restrictions for movement. This study found three-quarters of the students avoided going to crowded places during the week prior to answering this survey; however, only 33.3% were committed to wearing masks while being outdoors. Unfortunately, this unexpected low percentage contradicts the WHO recommendations, which encourage wearing the non-medical fabric masks in public places, especially when physical distancing of at least 1 m is not possible, such as public transport, shops, or in any other crowded environments [78].

Surprisingly, this behavior seems to be related to the participant’s gender and academic year. For example, undergraduate males in their second year are more likely to avoid wearing masks when they go to crowded places. A previous study undertaken in Jordan and Egypt, stated that men and late adolescents have a higher chance of participating in risk-taking behavior [74,75]. The higher risk-taking rates from the previously mentioned participants indicate the lack of commitment at the beginning of the pandemic. In this fashion, it is expected for this behavior to be present in some cities, and for the severity of the epidemic to be underestimated, which has led to an increase in the number of infected people in Palestine. 

Interestingly, Alzoubi et al. (2020) pointed out that no significant difference was apparent between the mean of KAP among medical and non-medical students [74]. In line with this study, no difference was found in the levels of KAP across the study programs, which can be explained by the fact that COVID-19 is a serious global threat about which the media is highly interested in providing comprehensive coverage, and the precautions that several governments committed to after WHO announced COVID-19 as a pandemic [23]. In contrast, Xiong et al. (2021) found that the Chinese medical students had significantly higher levels of knowledge and sense of awareness regarding the COVID-19 pandemic during its early stages compared to their non-medical peers [82].

### 4.1. Strengths

To the best of the authors’ knowledge, this is the first study to analyze the information sources and the information-seeking strategies among the Palestinian population whose humanitarian struggle was further complicated by the COVID-19 pandemic. The target population of this study was university students who are broadly viewed as the upper echelon of health literacy among their friends and social circles; therefore, their health-related awareness and behaviors are of the utmost importance for health promotion efforts as they can be either facilitators or barriers for public health messaging. The COVID-19-related infodemic is—supposedly—imposing greater risks on the youth population because of their high degree of access to social media platforms, where user-generated content can perfectly normalize and disseminate misinformation and fake news.

### 4.2. Limitations

The first limitation of this study is its dependence on the self-reported outcomes, which make it prone to both information and recall biases. The second limitation is the imbalance in the ratio of female to male respondents, which can be justified by the fact that this study aimed to recruit a nationally representative sample. The third limitation is that overlapping knowledge resources were not identifiable nor distinguishable in this study, as the same piece of information may reach the recipient through various platforms multiple times. Finally, as a cross-section study, it was not possible to follow up the trends of students’ information-seeking patterns and their COVID-19-related KAP, which were reported to be rapidly changing over the time.

### 4.3. Implications

The findings of this study imply that social media platforms are of practical value for health promotion strategies that target the young- and middle-age adult population. The information sources were found to affect the surveyed students’ attitudes towards COVID-19 and their practices including protective behaviors. This underlies the need for health literacy leverage, especially in conflict zones as a supportive strategy for the fragile health systems. These results call upon enhancing the cooperation between public health authorities in Palestine and universities in order to create awareness programs about the pandemics or other health emergencies through the educational curricula.

## 5. Conclusions

This study provided an insight into the information sources used by Palestinian university students, the sources they trusted, and the information formats they preferred. These results may help public health authorities to locate the information sources used by university students, and which thus should be targeted. Efforts should be undertaken to recommend healthcare workers as credible information sources. In this way, they will be able to prevent the spread of misleading information and provide high-quality information, especially within unconventional settings such as refugee camps. 

## Figures and Tables

**Table 1:** Information sources used by Palestinian students during the COVID-19 outbreak, May 2020.

Category	Source
Official Sources	World Health Organization (WHO)
	Palestinian Ministry of Health (MoH)
Scientific Sources	Scholarly Articles
	Healthcare Workers
Community Sources	Friends and Family Members
	Lecturers
	Social Media
Media Sources	Television (TV)
	Newspapers including news websites

**Table 2:** Demographic characteristics of the participating students, May 2020 (*n* = 951).

Variable	Outcome	Frequency	Percentage
Age	18–20 years	462	48.6%
	21–23 years	443	46.6%
	24–26 years	39	4.1%
	>26 years	7	0.7%
Gender	Female	664	69.8%
	Male	287	30.2%
Social Status	Single	919	96.6%
	Married	27	2.8%
	Divorced	4	0.4%
	Widow/er	1	0.1%
Study Field	Healthcare	337	35.4%
	Non-healthcare	614	64.6%
Study Year	1st Year	158	16.6%
	2nd Year	201	21.1%
	3rd Year	222	23.3%
	4th Year	236	24.8%
	5th Year	85	8.9%
	Other	49	5.2%
Residence	City	549	57.7%
	Village	337	35.4%
	Camp	65	6.8%
Monthly Household Income	<1000 ILS	71	7.5%
	1000–1999 ILS	111	11.7%
	2000–2999 ILS	160	16.8%
	3000–3999 ILS	177	18.6%
	4000–4999 ILS	108	11.4%
	5000–5999 ILS	132	13.9%
	≥5999 ILS	192	20.2%

**Table 3:** Responses of the participating Palestinian students to the COVID-19-related knowledge subscale, May 2020 (*n* = 951).

Item	Outcome	Frequency	Percentage
The main clinical symptoms of COVID-19 are fever, tiredness, sore throat, and dry cough.	Yes	923	97.1%
	No	14	1.5%
	I don’t know	14	1.5%
Some people become infected with SARS-CoV-2 but don’t develop any symptoms and don’t feel sick.	Yes	862	90.6%
	No	45	4.7%
	I don’t know	44	4.6%
It is not necessary for children and young adults to take measures to prevent the infection by SARS-CoV-2.	Yes	34	3.6%
	No	35	3.7%
	I don’t know	882	92.7%
SARS-CoV-2 can be transmitted through mosquito bites.	Yes	55	5.8%
	No	305	32.1%
	I don’t know	591	62.1%
SARS-CoV-2 cannot be transmitted in areas with hot and humid climates.	Yes	121	12.7%
	No	250	26.3%
	I don’t know	580	61%
Persons with COVID-19 are only infectious when they are showing symptoms.	Yes	91	9.6%
	No	77	8.1%
	I don’t know	783	82.3%
SARS-CoV-2 can transmit from through small droplets which are secreted when an infected person coughs or exhales.	Yes	896	94.2%
	No	35	3.7%
	I don’t know	20	2.1%
One of the best ways to protect yourself is to wash your hands frequently with soap or clean them with an alcohol-based hand rub.	Yes	910	95.7%
	No	18	1.9%
	I don’t know	23	2.4%
Physical distancing and treatment of people who are infected with SARS-CoV-2 are effective ways to reduce the spread of the virus.	Yes	917	96.4%
	No	14	1.5%
	I don’t know	20	2.1%
Older persons and persons with pre-existing medical conditions seem to develop serious illness more often than others.	Yes	885	93.1%
	No	46	4.8%
	I don’t know	20	2.1%

**Table 4:** Responses of the participating Palestinian students to the COVID-19-related attitudes subscale, May 2020 (*n* = 951).

Item	Outcome	Frequency	Percentage
Are you concerned about the impact that this outbreak will have on your community?	Yes	778	81.8%
	No	145	15.2%
	Not Sure	28	2.9%
Do you think that COVID-19 had been contained and will soon be over?	Yes	325	34.2%
	No	447	47%
	Not Sure	179	18.8%
Do you think the Palestinian Public Health Service was well prepared for the COVID-19 pandemic?	Yes	290	30.5%
	No	480	50.5%
	Not Sure	181	19%

**Table 5:** Responses of the participating Palestinian students to the COVID-19-related practices.subscale, May 2020 (*n* = 951).

Item	Outcome	Frequency	Percentage
Which of the following describes your current behavior?	I am continuing to socialize in public spaces (*code* = 0)	28	2.9%
	I do not know (*code* = 1)	16	1.7%
	I am continuing to socialize in public spaces but less often (*code* = 2)	92	9.7%
	I am not going to public spaces, but I am socializing with my friends or family in my or their homes (*code* = 3)	229	24.1%
	I am not going to public spaces, and I am socializing with my friends and family less often (*code* = 4)	375	39.4%
	I am not going to public spaces, and I am not socializing with friends or family (*code* = 5)	211	22.2%
In the past week, have you gone to any crowded places?	Yes	202	21.2%
	No	723	76%
	I prefer not to answer	26	2.7%
In the past week, have you worn a mask when leaving home?	Yes	317	33.3%
	No	572	60.1%
	Not Sure	62	6.5%

**Table 6:** Groups of information sources and wearing masks while being outdoors, May 2020 (*n* = 951).

Information Sources Groups	Frequency	Percentage	Knowledge Score		Did You Wear a Mask upon Leaving Your House Last Week?		
			*µ*	*SD*	Yes (%)	No (%)	Not Sure (%)
C	154	16.2%	8.5	1.6	23%	73%	5%
O	124	13%	8.6	1.3	31%	61%	7%
Sc	17	1.8%	8.6	1.4	35%	59%	6%
T	14	1.5%	8.6	2.8	43%	50%	7%
O + C	147	15.5%	8.8	1.6	24%	67%	8%
O + Sc	80	8.4%	9.1	1.3	50%	44%	6%
C + T	20	2.1%	7.8	2.7	30%	70%	0%
O + T	16	1.7%	8.4	1.5	50%	50%	0%
Sc + C	14	1.5%	8.5	1.1	29%	71%	0%
Sc + T	1	0.1%	8.0	-	0%	100%	0%
O + Sc + C	142	14.9%	8.8	1.3	39%	51%	10%
O + C + T	107	11.3%	8.5	1.5	29%	66%	5%
O + Sc + T	12	1.3%	8.5	1.9	58%	42%	0%
Sc + C + T	7	0.7%	8.9	1.5	43%	57%	0%
O + Sc + C + T	94	9.9%	8.8	1.4	44%	49%	7%
Other	2	0.2%	7.5	2.1	0%	50%	50%

O = official sources; Sc = scientific sources; C = community sources; T = traditional media sources.

**Table 7:** Information-seeking strategies and attitudes towards COVID-19 among Palestinian students, May 2020. (*n* = 951).

Variable	Outcome	Do You Think That COVID-19 Has Been Contained and Will Soon Be Over?		
		Yes (%)	No (%)	Not Sure (%)
Trusted Source	Scholarly Articles	33%	47%	20%
	WHO	31%	50%	18%
	MoH	43%	39%	18%
	Television	60%	40%	0%
	Family, Friends or Lecturers	60%	40%	0%
	Social Media	45%	30%	25%
	Healthcare Workers	33%	44%	23%
	Newspapers	100%	0%	0%
	Other	33%	50%	17%
Fact-checking Method	Official Sources	28%	52%	20%
	Healthcare Workers	44%	42%	14%
	Social Media	45%	39%	16%
	Searching Web	39%	47%	14%
	Family or Friends	44%	28%	28%
	Not Sure	42%	36%	23%
	Other	36%	45%	18%
Media Format	Video	36%	44%	20%
	Video and Text	67%	33%	0%
	Text	34%	48%	18%
	Photo	23%	56%	21%
	Voice	50%	33%	17%
	Charts	19%	59%	22%
	Other	0%	100%	0%
	Video, Text, Photos or Charts	50%	50%	0%

## Data Availability

The data that support the findings of this study are available from the corresponding author upon reasonable request.
